# Mild pharmacological inhibition of mitochondrial complex I restores synaptic density in hippocampus of APP/PS1 mice assessed by 3D EM reconstruction

**DOI:** 10.1002/alz.086562

**Published:** 2025-01-09

**Authors:** Noah Keller, Trace Christensen, Eugenia Trushina

**Affiliations:** ^1^ Department of Neurology, Mayo Clinic, Rochester, MN USA; ^2^ Microscopy and Cell Analysis Core Facility, Mayo Clinic, Rochester, MN USA; ^3^ Department of Molecular Pharmacology and Experimental Therapeutics, Mayo Clinic, Rochester, MN USA

## Abstract

**Background:**

Alzheimer’s disease (AD) is an age‐dependent neurodegenerative disorder with limited treatment options. As it progresses, synapse degeneration is the most important feature contributing to cognitive dysfunction. Mitochondria supply synapses with ATP for neurotransmitter release and vesicle recycling and buffer calcium concentrations. In AD, mitochondrial function is disrupted leading to energetic stress and synapse degeneration. We have identified a novel therapeutic strategy that blocks neurodegeneration and protects cognitive function in multiple mouse models of AD (amyloid and tau pathologies) via mild pharmacological inhibition of mitochondria complex I, which results in the activation of adaptive stress response and subsequent improvement of mitochondrial function.

**Method:**

Analysis of mitochondrial morphology and synaptic density was done in the hippocampal CA1 tissue from APP/PS1 and non‐transgenic (NTG) mice treated with vehicle or mild complex I inhibitor CP2 for 14 months starting after the development of cognitive dysfunction and neurodegeneration. A serial block‐face scanning electron microscopy (SBF‐SEM) and three‐dimensional (3D) EM reconstructions were employed to determine the synaptic density and mitochondrial morphology and distribution associated with different dendritic spine types.

**Result:**

We demonstrate that APP/PS1 mice have significantly reduced synaptic density in the hippocampus compared to the NTG littermates. CP2 treatment restores synaptic density to the levels observed in NTG mice evident by the increase in the number of synaptic densities visible in the electron micrographs. Mitochondrial morphology and association with synapses are currently being investigated.

**Conclusion:**

Our data are consistent with previously conducted electrophysiology recording in acute brain slices from APP/PS1 and NTG mice treated with vehicle or CP2, demonstrating restoration of long‐term potentiation (LTP) after chronic CP2 treatment. Additionally, it suggests that 3D EM‐based assessment of synaptic density could serve as a surrogate marker for LTP and cognitive function useful for drug discovery.

